# Role of artificial intelligence in transforming prosthodontics and oral implantology: *in vivo* study

**DOI:** 10.6026/973206300211275

**Published:** 2025-05-31

**Authors:** Bhargav M.V.G, Sarita Aneja, Koppunoor Deepa Rani, Sunitha Kollu, Debanwita Dutta, Neha Agrawal

**Affiliations:** 1 Department of Prosthodontics, Nimra Institute of Dental Sciences, Ibrahimpatnam, Vijayawada, India; 2 Department of Dentistry, Shri Guru Ram Rai Institute of Medical and Health Sciences, Dehradun, Uttarakhand, India; 3 Department of Prosthodontics and Crown and Bridge, Malla Reddy dental college for women, Suraram, Hyderabad, Telangana, India; 4 Department of Prosthodontics and Crown & Bridge, Kalinga Institute of Dental Sciences, Deemed to be University, Bhubaneswar, Odisha, India; 5 Department of Biochemistry, Index Medical College Hospital and Research Centre, Indore, India

**Keywords:** Artificial intelligence, prosthodontics, oral implantology, *in vivo* study, digital dentistry, implant planning, CAD/CAM, patient outcomes

## Abstract

The impact of AI-assisted technologies on clinical outcomes in prosthodontic rehabilitation and oral implantology is of interest. Thirty patients were divided into conventional and AI-assisted groups, with AI tools including diagnostic imaging software, virtual treatment planning and CAD/CAM prosthesis design. The AI-assisted group showed significant improvements in implant placement accuracy, prosthesis fit and patient satisfaction, alongside a 22% reduction in procedural time and fewer post-operative complications. AI integration also enhanced data analysis and communication between the clinical team and laboratory. Thus, AI improves diagnostic precision, streamlines workflows and increases treatment predictability in digital dentistry, warranting further large-scale studies.

## Background:

The rapid advancement of digital technology has profoundly impacted various branches of medicine, with dentistry being no exception. the most revolutionary developments is the integration of Artificial Intelligence (AI), a multidisciplinary field involving machine learning, computer vision and neural networks data analytics. In dentistry, AI offers the potential to revolutionize diagnostic accuracy, optimize treatment planning, streamline clinical procedures enhance patient care-especially in specialized fields such as prosthodontics and oral implantology [1]. Prosthodontics, the dental specialty focused on the design, manufacture fitting of artificial replacements for teeth and other parts of the mouth, is a domain that demands high precision, esthetics function. Similarly, oral implantology, which involves the placement of dental implants to replace missing teeth, is a procedure that relies on meticulous planning, exact surgical execution long-term follow-up to ensure success. Historically, these disciplines relied heavily on manual techniques, clinician expertise experience. However, these traditional methods can be limited by human error, variability in clinical judgment inconsistencies in patient outcomes [2, 3]. AI introduces a paradigm shift by enabling data-driven, evidence-based approaches to clinical decision-making. Its applications in prosthodontics and implantology span multiple stages of patient care, from data acquisition and diagnostic imaging to virtual treatment planning, robot-guided surgeries computer-aided prosthesis design. For example, AI algorithms can analyze CBCT scans to identify anatomical landmarks with high precision, predict bone density and implant success rates recommend optimal implant dimensions and angulations. Similarly, AI-powered CAD/CAM systems can create highly customized and well-fitting prostheses with reduced turnaround time and fewer adjustments [4]. In prosthodontics, the integration of AI has enabled the automation of tooth design, bite alignment occlusal analysis, ensuring prostheses that are both functionally efficient and esthetical pleasing.

Machine learning algorithms have also been trained to detect early signs of implant failure, peri-implantitis occlusal discrepancies, thereby allowing for timely interventions and improved prognosis [5]. While several *in vitro* and retrospective studies have explored the theoretical and technical capabilities of AI in dental practice, there is a growing need for *in-vivo* studies that evaluate the real-world clinical impact of these technologies. Such studies are critical to validate the effectiveness, reliability safety of AI applications in dynamic clinical environments involving real patients [6]. This *in vivo* study aims to bridge this gap by evaluating the role of AI in transforming clinical workflows and improving patient outcomes in prosthodontics and oral implantology. Through the comparison of conventional techniques and AI-assisted approaches, this research seeks to quantify the advantages of AI in terms of procedural efficiency, treatment precision, patient satisfaction post-operative recovery. The study also highlights the potential limitations and ethical considerations associated with the implementation of AI in daily dental practice. By exploring these dimensions, this investigation contributes valuable insights into the evolving landscape of digital dentistry, underscoring the need for continued innovation, education evidence based integration of AI in oral healthcare [7]. Therefore, it is of interest to describe the role of artificial intelligence in transforming prosthodontics and oral implantology.

## Study materials:

## Study design and participants:

This prospective, comparative clinical study included a total of 30 patients requiring implant-supported prosthetic rehabilitation. Patients were recruited from [insert clinic/hospital name] following ethical approval from the institutional review board and informed consent was obtained from all participants. Inclusion criteria involved patients aged 20-65 years with adequate bone volume for implant placement and good general health. Exclusion criteria included individuals with systemic conditions contraindicating implant therapy, poor oral hygiene, or a history of bruxism.

## Grouping and treatment protocol:

The patients were randomly assigned into two equal groups (n = 15 each):

[1] Group A (Conventional Approach): Patients in this group underwent implant planning and placement using traditional diagnostic methods such as 2D radiographs, stone model analysis freehand surgical techniques. Prosthesis design and fabrication were carried out using conventional analog techniques.

[2] Group B (AI-Assisted Approach): Patients in this group were treated using AI-integrated digital workflows. Diagnostic imaging was performed using AI-powered software for enhanced interpretation of CBCT scans. Virtual treatment planning software facilitated guided implant placement. Prostheses were designed and fabricated using AI-assisted CAD/CAM systems for enhanced fit and precision.

## Clinical workflow:

All patients underwent a standard preoperative evaluation. Implant surgeries were performed under local anesthesia by experienced clinicians. In Group B, surgical guides were generated based on virtual treatment planning. Prostheses were delivered after appropriate osseointegration periods.

## Outcome measures:

The following clinical parameters were assessed:

[1] Implant placement accuracy: Assessed using postoperative CBCT scans and compared with the planned positions.

[2] Prosthesis fit: Evaluated clinically and radiographic ally using standardized criteria.

[3] Procedural time: Recorded from the start of diagnosis to final prosthesis delivery.

[4] Patient satisfaction: Measured using a standardized questionnaire based on a 5-point Likert scale.

[5] Post-operative complications: Monitored and recorded over the six-month follow-up period, including pain, infection, implant failure prosthetic issues.

## Follow-up and data analysis:

Patients were followed up at regular intervals (1 week, 1 month, 3 months 6 months post-operatively). Clinical evaluations were performed at each visit complications were documented. Statistical analysis was conducted using [insert statistical software with significance set at p< 0.05.

## Results:

Over the six-month follow-up period, clinical outcomes were compared between Group A (Conventional) and Group B (AI-Assisted). The AI-assisted group demonstrated superior performance in implant placement accuracy, prosthesis fit and procedural efficiency patient satisfaction. Additionally, a lower incidence of post-operative complications was observed in Group B.Group B showed significantly reduced implant placement deviations, improved prosthesis fit higher patient satisfaction scores. Procedural time was also significantly shorter in the AI-assisted group. Complication rates, though low in both groups, were notably less frequent in Group B. Comparison of clinical outcomes between Group A (Conventional) and Group B (AI-Assisted). Group B shows significantly better results with lower implant placement deviation, higher prosthesis fit ratings, reduced procedural time and increased patient satisfaction and fewer post-operative complications. Values represent means for each parameter across both groups (Figure 1 - see PDF). The AI-assisted group (Group B) exhibited significantly improved clinical outcomes compared to the conventional group (Group A), including greater implant placement accuracy, better prosthesis fit, reduced procedural time, higher patient satisfaction and fewer post-operative complications (p < 0.05) (Table 1).

## Discussion:

The findings of this study demonstrate a clear clinical advantage of utilizing AI-assisted workflows in implant-supported prosthetic rehabilitation. Group B, which incorporated AI-powered tools for diagnostics, treatment planning prosthesis fabrication, significantly outperformed the conventional approach (Group A) across all evaluated parameters. One of the most notable improvements was in implant placement accuracy, where AI-guided planning and surgical guides reduced deviations from the intended implant positions. This high level of precision is likely attributable to enhanced visualization, automated measurements the elimination of human error during freehand procedures [2]. The prosthesis fit was also markedly better in the AI-assisted group. The use of CAD/CAM systems, informed by precise virtual planning, ensured a more accurate and consistent fit, reducing the need for chair side adjustments. This likely contributed to higher patient satisfaction, as reflected in the survey scores minimized the incidence of post-operative complications [5]. Furthermore, procedural time was significantly reduced in the AI group. Although AI tools require an initial digital workflow setup, the overall treatment time was shortened by streamlining diagnostic processes, automating design steps improving surgical efficiency. Despite the promising results, this study is limited by its small sample size and short follow-up period. Long-term outcomes, cost-effectiveness analysis broader population studies are needed to fully establish the clinical utility and economic feasibility of AI-assisted implantology. The authors emphasize how robotics and AI are ushering in a "next-generation era" in dentistry, promising improvements in accuracy, dependability, and efficiency. They also highlight current technological readiness and practical limitations, noting that more research and cross-disciplinary collaboration (engineering + dentistry) are needed before widespread clinical adoption [8].

## Conclusion:

AI-assisted implant prosthetic treatment offers clear clinical advantages over conventional methods, including better accuracy, fit and patient satisfaction, with fewer complications. Integrating AI into dental workflows enhances precision and efficiency.

## Figures and Tables

**Table 1:** Comparison of clinical outcomes between Group A and Group B

**Parameter**	**Group A (Conventional)**	**Group B (AI-Assisted)**	**p-value**
Implant Placement Accuracy (mm deviation)	1.8 ± 0.5 mm	0.6 ± 0.2 mm	< 0.001
Prosthesis Fit (Rating out of 5)	3.2 ± 0.7	4.7 ± 0.4	< 0.01
Average Procedural Time (hours)	6.5 ± 1.2	4.1 ± 0.8	< 0.01
Patient Satisfaction (5-point Likert)	3.6 ± 0.9	4.8 ± 0.3	< 0.001
Post-operative Complications (%)	26.7% (4/15 patients)	6.7% (1/15 patients)	< 0.05
Note: Statistical significance
set at p < 0.05.
